# Rapid inverse radiative transfer solver for multiparameter spectrophotometry without integrating sphere

**DOI:** 10.1117/1.JBO.29.S1.S11508

**Published:** 2024-01-02

**Authors:** Jiahong Jin, Zachary D. Jones, Jun Q. Lu, Xin-Hua Hu

**Affiliations:** a Hunan Institute of Science and Technology, Institute for Advanced Optics, Yueyang, China; b East Carolina University, Department of Physics, Greenville, North Carolina, United States; c Hunan Institute of Science and Technology, School of Physics and Electronic Science, Yueyang, China

**Keywords:** inverse scattering problems, Monte Carlo modeling, light scattering and absorption, turbid materials, spectrophotometry

## Abstract

**Significance:**

Multiparameter spectrophotometry (MPS) provides a powerful tool for accurate characterization of turbid materials in applications such as analysis of material compositions, assay of biological tissues for clinical diagnosis and food safety monitoring.

**Aim:**

This work is aimed at development and validation of a rapid inverse solver based on a particle swarm optimization (PSO) algorithm to retrieve the radiative transfer (RT) parameters of absorption coefficient, scattering coefficient and anisotropy factor of a turbid sample.

**Approach:**

Monte Carlo (MC) simulations were performed to obtain calculated signals for comparison to the measured ones of diffuse reflectance, diffuse transmittance and forward transmittance. An objective function has been derived and combined with the PSO algorithm to iterate MC simulations for MPS.

**Results:**

We have shown that the objective function can significantly reduce the variance in calculated signals by local averaging of an inverse squared error sum function between measured and calculated signals in RT parameter space. For validation of the new objective function for PSO based inverse solver, the RT parameters of 20% Intralipid solutions have been determined from 520 to 1000 nm which took about 2.7 minutes on average to complete signal measurement and inverse calculation per wavelength.

**Conclusion:**

The rapid solver enables MPS to be translated into easy-to-use and cost-effective instruments without integrating sphere for material characterization by separating and revealing compositional profiles at the molecular and particulate scales.

## 1 Introduction

Conventional spectrophotometers are probably the most commonly used type of instruments for material analysis by determination of absorbance A or attenuation coefficient $\mu_{t}$ as a function of wavelength λ for a given sample. For turbid materials including biological tissues, additional optical parameters are needed to characterize molecular composition and particle sizes on scales close to λ by their ability to absorb and scatter light. One paradigm is to retrieve these parameters by the radiative transfer (RT) theory,^1^ and its spectroscopic implementation is termed multiparameter spectrophotometry (MPS). The RT theory quantifies light–matter interaction by the parameters of absorption coefficient $\mu_{a}$ and scattering coefficient $\mu_{s}$ in addition to a single-scattering phase function p(θ,ϕ) with θ and ϕ as the polar and azimuthal scattering angles. MPS thus requires measurement of multiple light scattering signals to solve multiple inverse scattering problems (ISPs) at selected values of λ that remains challenging for highly turbid samples. To reduce complexity, p(θ,ϕ) of an unknown sample is often modeled by an analytical function of p_HG_(cosθ) proposed by Heyney and Greenstein under the assumption of axially symmetric scattering.^1^^–^^3^ Alternative model functions have been studied for p(θ,ϕ) but $p_{\mathrm{HG}}(\cos\;\theta )$ is the preferred one with a form fully specified by an anisotropy factor g as the mean value of cos θ. By choosing p_HG_ as the phase function, MPS is to measure multiple light scattering signals and determine inversely the RT parameters as function of at λ. These parameters can be expressed as a vector of $P(\lambda )=(\mu_{a},\mu_{s},g)$.

A widely used approach of MPS is to measure three signals of collimated transmittance $T_{c}$, hemispherically integrated diffuse reflectance $R_{\mathrm{dh}}$ and transmittance $T_{\mathrm{dh}}$ with one or two integrating sphere.^3^^–^^11^ If only one integrating sphere is used, signals need to be measured in two steps with one for $R_{\mathrm{dh}}$ and another one for $T_{\mathrm{dh}}$ with $T_{c}$ acquired in either step. To solve the ISPs, one first derives $\mu_{t}$ ($=\mu_{a}+\mu_{s}$) from $T_{c}$ by the Beer-Lambert law followed with retrieval of $\mu_{s}$ and g from $R_{\mathrm{dh}}$ and $T_{\mathrm{dh}}$ using different methods of forward modeling guided by gradient descent based inverse solver. The modeling methods include numerically solving the RT boundary-value problem, adding-doubling algorithm or Monte Carlo (MC) simulations. Despite its popularity as a research tool, the need for two integrating spheres or two steps of signal measurement makes it difficult to translate this approach into an easy-to-use instrument like a conventional spectrophotometer. In addition, the use of integrating sphere limits severely the accessibility of the approach to non-specialists due to time-consuming sample assembly and system maintenance. Other approaches without integrating sphere have been investigated such as goniometric measurement and detection of $T_{c}$ and non-hemispherical $R_{d}$ and $T_{d}$ at fixed angles with MC based inverse algorithms.^12^^,^^13^

We have previously shown that the RT parameter vector P can be uniquely determined from three simultaneously measured signals of non-hemispherical diffuse reflectance $R_{d}$, diffuse transmittance $T_{d}$ and forward transmittance $T_{f}$ without integrating sphere.^14^^,^^15^ MC simulations are performed to accurately calculate signals as $R_{\mathrm{dc}},T_{\mathrm{dc}}$, and $T_{\mathrm{fc}}$ and repeated to match the measured ones. A gradient descent algorithm has been developed to guide iteration in the RT parameter space to minimize an objective function δ(P) defined as the sum of squared percentage differences between the measured and calculated signals. An ISP at λ is deemed as solved with $P_{s}$ when $\delta (P_{s})\leq \delta_{th}$ in which $\delta_{\mathrm{th}}$ represents a threshold based on the experimental errors in signal measurement. The inherent statistical variance in the calculated signals, however, makes it difficult to accurately solve ISPs for highly turbid and optically thick samples by gradient decent despite the existence of a unique solution.^16^ Specifically, sizable regions of small δ values exist in the RT parameter space of **P** for these ISPs of large scattering albedo $a(=\mu_{s}/\mu_{t})$ and optical thickness $\tau (=\mu_{t}D)$ with D as sample thickness. In such regions, values of δ(P) fluctuate considerably due to the variance of MC simulations that often lead to errors in the solution given by $P_{s}$. To solve these challenging ISPs for highly turbid samples, one needs to either significantly increase the number of photons in MC simulations for variance reduction or search manually by contour analysis of δ(P) distributions in the RT parameter space. Either way gives rise to high computational cost and prevents rapidly solving ISPs for MPS.^16^ In this report, we present a rapid inverse solver for MPS based on a particle swarm optimization (PSO) algorithm with a novel objective function $\rho_{1/\delta }(P)$ through local averaging in the space of P to determine $P_{s}(\lambda )$.^17^ The function $\rho_{1/\delta }(P)$ significantly reduces the effect of MC simulation variance in calculated signals on inverse calculation by PSO and computation time to solve ISPs by reducing the number of photons in MC simulations. Our validation results with 20% intralipid samples demonstrate that $P_{s}(\lambda )$ can be retrieved rapidly between 520 and 1000 nm by executing MC simulations on one GPU board.

## 2 Materials and Methods

### 2.1 Signal Measurement by MPS

**Fig. 1:**
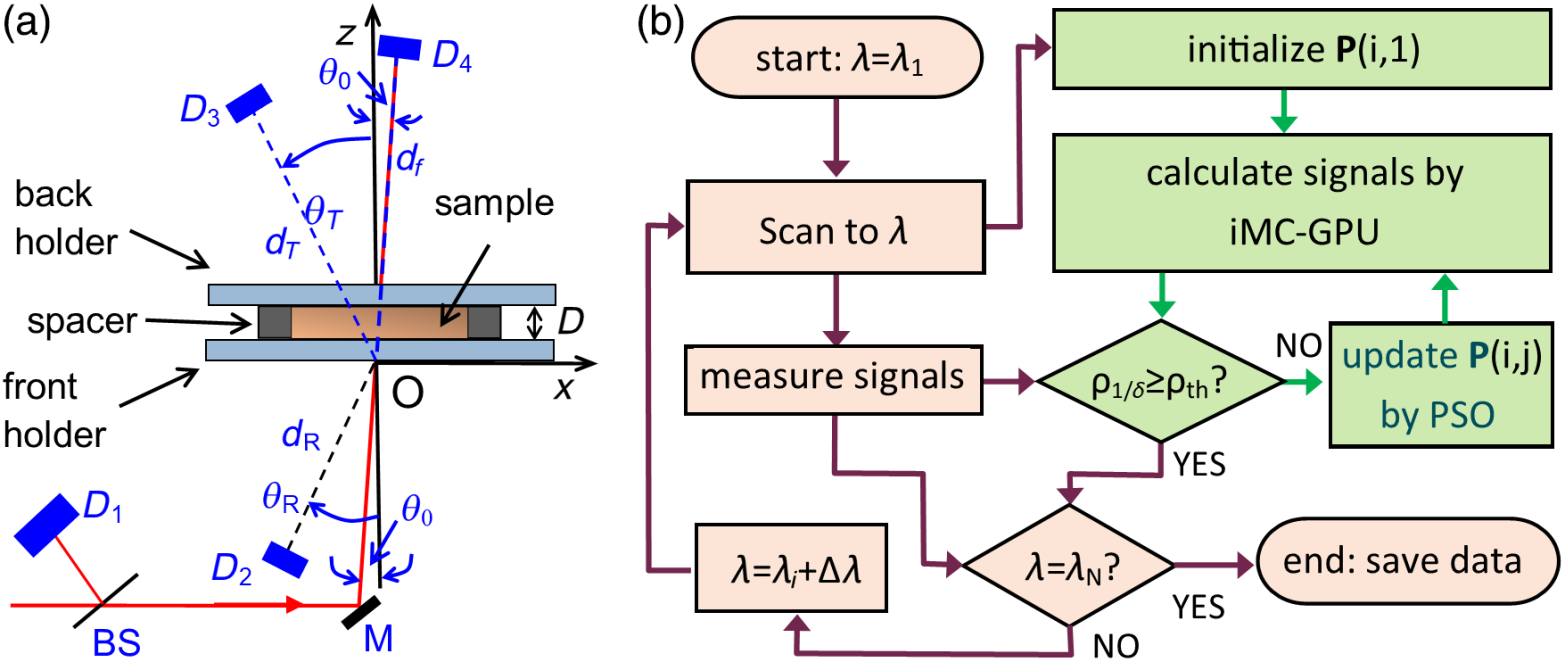
(a) Configuration of signal detection with incident beam indicated by the red line: D: sample thickness; $D_{1}$: for monitoring $I_{0}$; M: mirror; $D_{2}$ to $D_{4}$: for measurement of $I_{\mathrm{Rd}},I_{\mathrm{Td}}$, and $I_{\mathrm{Tf}}$; $\theta_{0}$: angle of incident beam from z-axis; $d_{R},d_{T}$, and $d_{f}$: distance between the origin and front center of $D_{2}$, $D_{3}$, and $D_{4}$; and $\theta_{R},\theta_{T}$, and $\theta_{f}$: angle of sensor surface normal (blue dash lines) of *D*_2_, *D*_3_, and *D*_4_ from the *z*-axis. (b) Work-flow chart of signal measurement in brown boxes and inverse calculation in green boxes.

An experimental system has been constructed to measure $R_{d}(\lambda )$, $T_{d}(\lambda )$, and $T_{f}(\lambda )$ for validation of the new inverse solver with the signal detection configuration shown in Fig. 1(a). The details of the experimental system were previously reported.^18^ Briefly, a xenon light source (XL1-175-A, WavMed Technologies Corp.) and a monochromator (CM110, CVI Corp.) are employed to produce a monochromatic beam with *λ* adjustable between 520 and 1000 nm in steps of 20 nm and bandwidths around 5 nm. The beam is modulated at a frequency of $f_{0}=370\;\mathrm{Hz}$ by a mechanical chopper (SR540, Stanford Research Systems) and incident on an assembly consisting of a turbid sample confined in a spacer ring between two glass slides. The intensity of the incident beam $I_{0}$ is monitored by a photodiode of $D_{1}$ (FDS1010, Thorlabs, Inc.). Three photodiodes (FDS100, Thorlabs, Inc.) of $D_{2}$ to $D_{4}$ are used to measure respectively $I_{\mathrm{Rd}}$ for diffusely reflected, $I_{\mathrm{Td}}$ for diffusely transmitted and $I_{\mathrm{Tf}}$ for forwardly transmitted light intensity. The current signals of photodiodes were amplified by an in-house built four-channel lock-in amplifier to obtain the measured signals of $R_{d}=I_{\mathrm{Rd}}/I_{0},T_{d}=I_{\mathrm{Td}}/I_{0}$ and $T_{f}=I_{Tf}/I_{0}$. Figure 1(a) shows the detection configuration for acquisition of measured signals from the sample assembly.

### 2.2 Signal Calculation by iMC

An in-house developed individual photon tracking MC (iMC) code was employed to calculate signals as $R_{dc},T_{dc}$ and $T_{fc}$ from given P for a phantom of the same shape as the sample inside a spacer between two glass slides by tracking N_0_ photons, which imports the parameters of sample size and detection configuration as input data.^16^^,^^19^^,^^20^ The code injects each of the N_0_ photons incident on the sample assembly and then tracks the photon once it transports inside a glass slide or sample until it is either absorbed inside the sample or escapes into air. The exit location and propagation direction of an escaping photon on a glass slide surface are used to determine if it hits a detector for detection. A counter associated with each detector records the number of detected photons as $N_{\mathrm{Rd}},N_{\mathrm{Td}}$, and $N_{\mathrm{Tf}}$ by the detector $D_{2},D_{3}$, and $D_{4}$, respectively. The above process repeats until the total number of injected photons reaches $N_{0}$ and the calculated signals are given by $R_{\mathrm{dc}}=N_{\mathrm{Rd}}/N_{0}$, $T_{\mathrm{dc}}=N_{\mathrm{Rd}}/N_{0}$ and $T_{\mathrm{fc}}=N_{\mathrm{Tf}}/N_{0}$. Because of independence in trajectory among the $N_{0}$ photons, the numbers of detected photons follow Poisson distributions.^21^ To estimate the variance in these photon numbers, one can draw a random number q of Poisson distribution with $f(q,q_{m})$ as probability mass function and $q_{m}$ as the mean. In the case of $N_{\mathrm{Rd}}$ calculated by an iMC simulation, $q_{m}$ equals to $N_{0}R_{\mathrm{dc}\infty }$ if distribution of $N_{\mathrm{Rd}}$ follows $f(N_{\mathrm{Rd}},q_{m})$ and $R_{\mathrm{dc}\infty }$ yields the variance-free value of $R_{\mathrm{dc}}$ by tracking “infinite” number of photons. One thus can use $f(q,q_{m})$ to quantify the effect of variance on calculated signals and optimize the objective function for variance reduction.

### 2.3 PSO Algorithm

A stochastic algorithm based on PSO has been developed to guide iMC simulations and solve for $P_{s}(\lambda )$ in the RT parameter space from measured signals by optimizing an objective function as shown in Fig. 1(b). An objective function quantifies the difference between measured signals and calculated ones obtained by given P(λ), and optimization of this function reduces the difference to solve for $P_{s}(\lambda )$ from an initial choice of P(λ). The PSO algorithm was chosen for its high efficiency and ability to perform global search or avoid local traps in the RT parameter space. A search proceeds through multiple threads in PSO, and the threads are represented by a swarm of “particles” with index i∈[1,I] and I as the number of threads or particles. Here, a particle i symbolizes a thread of positions in the RT parameter space along which it moves from P(i,j) to P(i,j+1) as^22^

$$P(i,j+1)=P(i,j)+v(i,j+1),\;v(i,j+1)=\chi \{w_{0}v(i,j)+w_{p}q(P_{p}(i)-P(i,j))+w_{g}q\prime (P_{g}-P(i,j))\},$$ (1)

where j∈[1,J]; J is the number of iterations; v is the particle’s velocity; q and q’ are random numbers of uniform distribution from 0 to 1; $P_{p}(i)$ is the particle-best position; $P_{g}$ is the swarm-best position; $w_{0},w_{p}$, and $w_{g}$ are weights of terms contributing to v(i,j+1); and χ is a damping constant to increase stability. A particle transits toward $P_{p}(i)$ and $P_{g}$ through completed iterations by Eq. (1) that respectively yields the best position of that particle and all particles for optimized objective function to reduce difference between calculated and measured signals. By setting values of $w_{p}$ and $w_{g}$ equal and large in comparison to $w_{0}$ in Eq. (1), for example, enables fast convergence of all particles from their best positions $P_{p}(i)$ towards $P_{g}$ on average as *j* increases. A region $\Gamma_{s}$ in the RT parameter space is designated as the search space and P(i,j+1) in Eq. (1) is set to P(i,j) if the former moves out of $\Gamma_{s}$. The search stops once the objective function at $P_{g}$ reaches a preset threshold or j exceeds J and current $P_{g}$ is saved as $P_{s}(\lambda )$ for output.

## 3 Results and Discussion

### 3.1 Effect of Variance in Calculated Signals on Solving ISPs

MPS requires solving one ISP for each value of λ from the measured signals. The first step is to perform forward calculations of signals from given $P=(\mu_{a},\mu_{s},g)$ by iMC simulations of light–matter interaction in the sample. The calculated signals can be normalized by respective measured signals and expressed as a vector of $S_{c}(P)=(R_{\mathrm{dc}}(P)/R_{d},$ $T_{\mathrm{dc}}(P)/T_{d},T_{\mathrm{fc}}(P)/T_{f})$. An inverse algorithm is to iterate iMC simulations toward the objective of making $S_{c}(P)$ as close to 1=(1,1,1) as possible. A function of squared error sum $\delta (P)=|S_{c}(P)-1{|}^{2}$ is often selected as the objective function to guide iteration and decide when stops. Ideally, $\delta (P_{s})$ vanishes for a perfect ISP solution given by $P_{s}$. In practice, one deems an ISP as solved if $\delta (P)\leq \delta_{th}$ with $\delta_{th}$ representing a threshold determined by the error level in signal measurement. To quantify the effect of variance in calculated signals by a stochastic iMC simulation, we define a fluctuation vector for calculated signals as

$$f_{c}=(\frac{R_{\mathrm{dc}}(P)}{R_{\mathrm{dc}\infty }(P)}-1,\frac{T_{\mathrm{dc}}(P)}{T_{\mathrm{dc}\infty }(P)}-1,\frac{T_{\mathrm{fc}}(P)}{T_{\mathrm{fc}\infty }(P)}-1),$$ (2)

where $R_{\mathrm{dc}\infty }$, $T_{\mathrm{dc}\infty }$, and $T_{\mathrm{fc}\infty }$ are the variance-free signals calculated by tracking “infinite” numbers of photons. It should be noted that $f_{c}$ is independent of P and one can express $S_{c}(P)$ by $f_{c}$

$$S_{c}(P)=(\frac{R_{\mathrm{dc}}(P)}{R_{d}},\frac{T_{\mathrm{dc}}(P)}{T_{d}},\frac{T_{\mathrm{fc}}(P)}{T_{f}})=S_{c\infty }(P)+S_{c\infty }(P)\circ f_{c},$$ (3)

with $S_{c\infty }(P)=(R_{\mathrm{dc}\infty }(P)/R_{d},T_{\mathrm{dc}\infty }(P)/T_{d},T_{\mathrm{fc}\infty }(P)/T_{f})$ and “o” denoting the vector form of component-wise product. Combining these results, δ(P) can now be written as

$$\delta (P)=(\frac{R_{dc}(P)}{R_{d}}-1{)}^{2}+(\frac{T_{dc}(P)}{T_{d}}-1{)}^{2}+(\frac{T_{dc}(P)}{T_{d}}-1{)}^{2}=\{S_{c}(P)-1\}\cdot \{S_{c}(P)-1\}\;=\delta_{f}(P)+2f_{c}\cdot \{S_{c\infty }(P)\circ (S_{c\infty }(P)-1)\}+|S_{c\infty }(P)\circ f_{c}{|}^{2},$$ (4)

where $\delta_{f}(P)=|S_{c\infty }(P)-1{|}^{2}$ is the variance-free value of δ(P). It is clear that $\delta_{f}(P_{s})=0$ if $P_{s}$ represents a “true” solution of ISP.

### 3.2 Comparison of Two Objective Functions for Variance Suppression

**Fig. 2:**
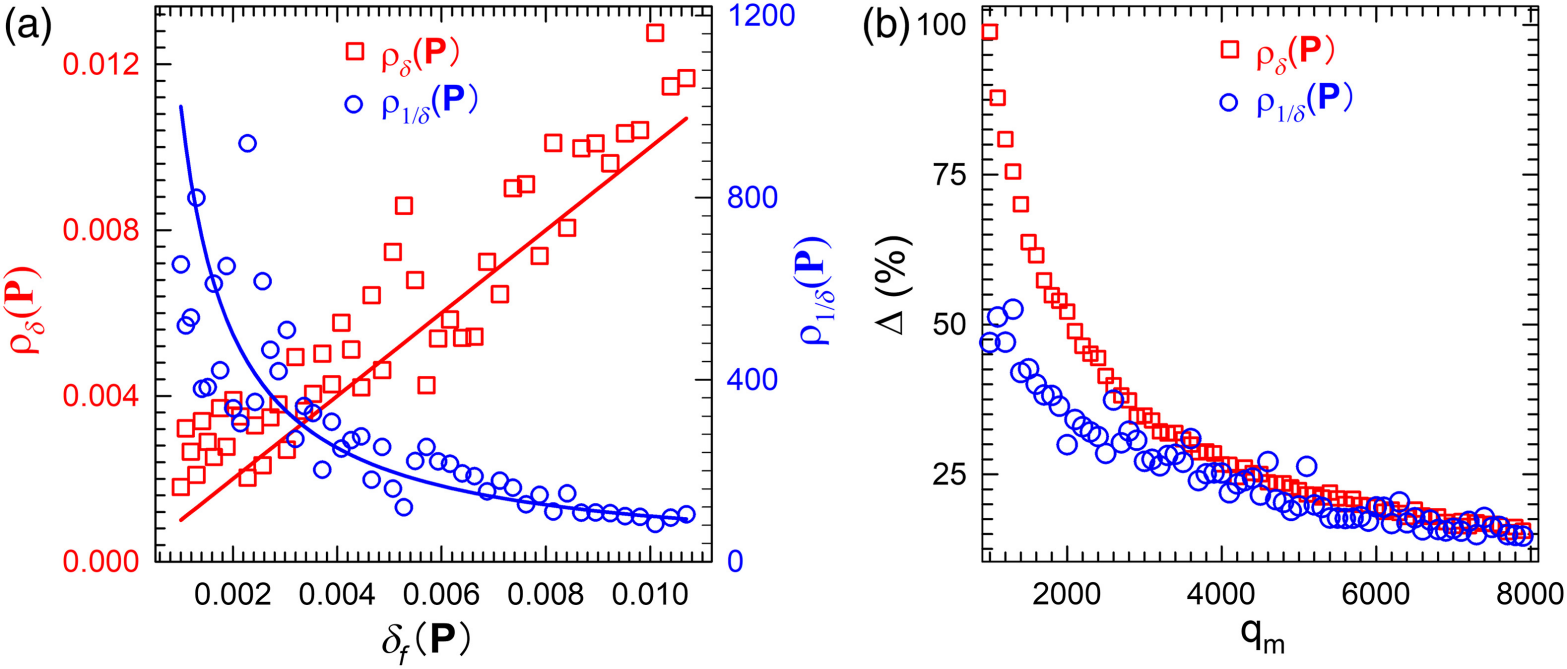
(a) The dependence of $\rho_{\delta }(P)$ and $\rho_{1/\delta }(P)$ on $\delta_{f}(P)$ with red line for $\delta_{f}(P)$ and blue line for $\delta_{f}(P{)}^{-1}$. (b) The mean values of relative differences $\Delta (\rho_{\delta })$ between $\rho_{\delta }(P)$ and $\delta_{f}(P)$ and $\Delta (\rho_{1/\delta })$ between $\rho_{1/\delta }(P)$ and $\delta_{f}(P{)}^{-1}$ against $q_{m}$ as the mean of Poisson distributions for calculated signals by tracking “infinite” photons.

Based on above analysis, local averaging on δ(P) in the RT parameter space was first employed to suppress variance and hence increase the stability of inverse solutions. We then compared two objective functions defined below on their abilities to further reduce the effect of variance

$$\rho_{\delta }(P)=\frac{1}{m_{P}}\underset{P\prime \in A_{P}}{\sum }\delta (P\prime ),\rho_{1/\delta }(P)=\frac{1}{m_{P}}\underset{P\prime \in A_{P}}{\sum }\frac{1}{\delta (P\prime )},$$ (5)

where $A_{P}$ is a set of $m_{P}$ vectors consisting of P and its neighbors on a 3D grid of P. The components of $f_{c}$ as defined in Eq. (2) can be obtained as $(q/q_{m})-1$ by drawing a random number q independently from $f(q,q_{m})$ and setting the $q_{m}$ values, related to $R_{\mathrm{dc}\infty },T_{\mathrm{dc}\infty }$, and $T_{\mathrm{fc}\infty }$, equal to focus on the effect of variance for simplicity. We further set $m_{P}=7$ with P and its nearest neighbors as the members of $A_{P}$ and assumed that all of them have the same value of $\delta_{f}(P)$. The values of $\rho_{\delta }(P)$ and $\rho_{1/\delta }(P)$ are plotted in Fig. 2(a) against $\delta_{f}(P)$ ranging from 0.1% to 1% with a solid line representing $\delta_{f}(P)$ or $\delta_{f}(P{)}^{-1}$. These results show clearly that $\rho_{1/\delta }(P)$ fluctuates significantly less than $\rho_{\delta }(P)$ for $\delta_{f}(P)$ between 0.6% and 1%, which is the typical range of $\delta_{th}$ set by the error level of measured signals.^16^

To gain further insight on fluctuation in calculated signals, the relative difference Δ between $\rho_{\delta }(P)$ and $\delta_{f}(P)$ or $\rho_{1/\delta }(P)$ and $\delta_{f}(P{)}^{-1}$ are calculated and averaged as

$$\Delta (\rho_{\delta })=\frac{1}{m_{\delta }}\overset{1.1\%}{\underset{\delta_{f}=0.1\%}{\sum }}|\frac{\rho_{\delta }(P)-\delta_{f}(P)}{\delta_{f}(P)}|,$$ (6)

and

$$\Delta (\rho_{1/\delta })=\frac{1}{m_{\delta }}\overset{1.1\%}{\underset{\delta_{f}=0.1\%}{\sum }}|\frac{\rho_{1/\delta }(P)-\delta_{f}(P{)}^{-1}}{\delta_{f}(P{)}^{-1}}|,$$ (7)

where $\delta_{f}$ is uniformly sampled between 0.1% and 1.1% in each sum and $m_{\delta }$ is the total number of $\delta_{f}$ samples in the sums. The sampled values of $\delta_{f}(P)$ correspond to the mean values of each signal term in $\delta_{f}(P)$ ranging from 1.8% to 6.1% since $\delta_{f}(P)$ is defined as the sum of squared relative differences of calculated and measured values on three signals. Figure 2(b) illustrates the dependence of the two Δ functions on $q_{m}$ values with $m_{\delta }$ set to 1000. As discussed before, $q_{m}$ represents the mean value of $f(q,q_{m})$ which is the probability mass function of Poisson distribution for modeling detected photon numbers, such as $N_{Rd}$ and related calculated signal $R_{dc}.$ Therefore, the ratio of $q_{m}/N_{0}$ equals to $R_{dc\infty }$ as the variance-free value of $R_{dc}$ in the above case and larger $q_{m}$ corresponds to larger $N_{0}$. The results in Fig. 2(b) demonstrate that use of $\rho_{1/\delta }(P)$ instead of $\rho_{\delta }(P)$ or δ(P) as the objective function can significantly reduce the variance of calculated signals for the same value of $N_{0}$. Alternatively, $N_{0}$ can be considerably reduced for the same variance to speed up forward calculations if $\rho_{1/\delta }(P)$ is adopted. For example, computational time of iMC simulations can be cut in half with $\rho_{1/\delta }(P$), instead of $\rho_{\delta }(P)$, as the objective function by tracking half of photons with similar variance.

### 3.3 Measurement of 20% Intralipid Sample

**Fig. 3:**
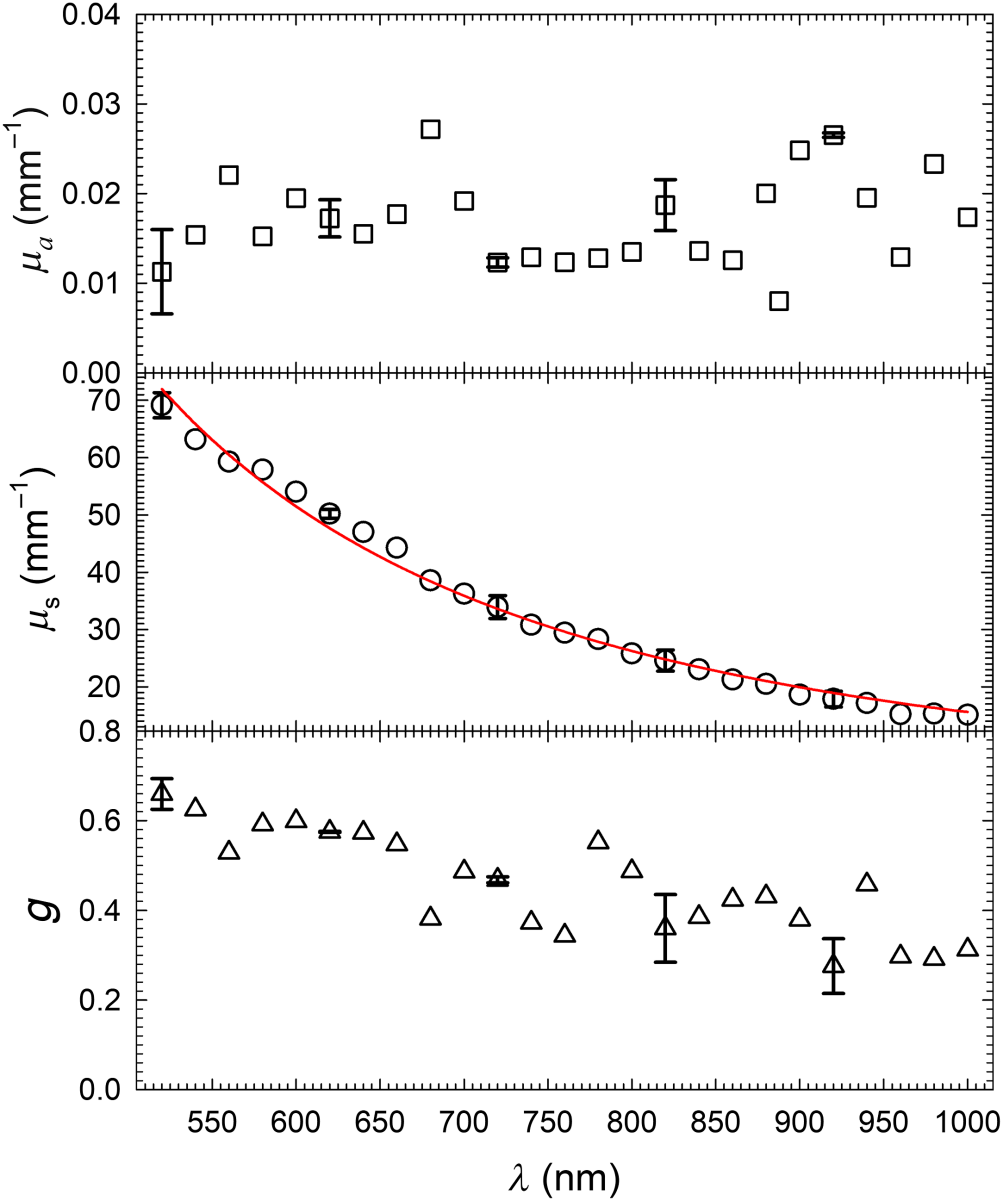
The wavelength dependence of RT parameters of one 20% intralipid sample of D=102  μm in thickness. The error bars were inversely determined from different combinations of the mean and standard deviation values of the measured signals shown in Fig. S1 in the Supplementary Material on five selected wavelengths. The red line is a power law fitting of $\mu_{s}=C\lambda^{-2.340}$ with $C=1.626\times 10^{8}$.

To validate the new approach, RT parameters of 20% intralipid (I141-100ML, Sigma-Aldrich) have been determined from three measured signals of $R_{d},T_{d}$, and $T_{f}$ for λ from 520 to 1000 nm in steps of 20 nm. Figure 3 presents the RT parameters of a sample with thickness D=102  μm obtained by the PSO based algorithm with $\rho_{1/\delta }(P)$ selected as the objective function. Signal measurement was repeated three times to obtain the mean values and standard deviations which are plotted in Fig. S1 in the Supplementary Material. Previously determined values of real refractive index $n_{r}(\lambda )$ of the 20% intralipid were used to obtain interpolated values in iMC simulations at the wavelengths of measurement for the calculated signals and $n_{r}(\lambda )$ is presented in Fig. S2 in the Supplementary Material.^23^ The same measurements were repeated on another sample of D=81  μm and the inversely determined RT parameters agree well with those in Fig. 3 within the experimental errors.

The search region $\Gamma_{s}$ for PSO based inverse algorithm at each wavelength was set between $1.00\times 10^{-4}$ and $2.00\;{\mathrm{mm}}^{-1}$ for $\mu_{a}$, 10.0 and $200\;{\mathrm{mm}}^{-1}$ for $\mu_{s}$, 0.10 and 1.00 for g. In additional to choosing I=27 for the particle number and J=100, we set $w_{0}=0.900$, $w_{p}=w_{g}=2.05$, and χ=0.730 for the PSO parameters defined in Eq. (1) for optimized performance of inverse calculations. The initial positions of P(i,1) for all particles were randomly distributed in each of nine equal partitions of $\Gamma_{s}$ to ensure globally optimized solutions of ISPs. We found that the convergence toward the final solution was not affected by the choice of P(i,1) under the above condition. After calculation of $\rho_{1/\delta }$ with P(i,1), iterations as defined in Eq. (1) were performed in $\Gamma_{s}$. Specifically, $P_{p}(i)$ was set as P(i,1) for particle i and $P_{g}$ as $P_{p}(i^{\prime })$ that has the largest $\rho_{1/\delta }$ value among all particles for j=1 to obtain v(i,2) and P(i,2) by Eq. (1). The search then continued as *j* increases to 2, 3,… and stopped when either $\rho_{1/\delta }(P_{g})$ exceeds $\rho_{th}$ for j≤J or j>J with $P_{g}$ saved as $P_{s}(\lambda )$ and output together with $\rho_{1/\delta }(P_{g})$ and $\delta (P_{g})$. The threshold $\rho_{\mathrm{th}}$ corresponds to the value of $\rho_{1/\delta }$ in Eq. (5) with $\delta_{\mathrm{th}}$ set to 0.8% and $m_{P}$ to 7.

It took about eight iterations on average to solve an ISP of $P_{s}(\lambda )$ from the measured signals of 20% intralipid sample. The total numbers of iMC simulations were found to be about 800 for solving each ISP if 27 particles were employed in PSO based search. Signal were calculated by iMC simulations in which $N_{0}$ was gradually increased from $5\times 10^{5}$ to $5\times 10^{6}$ as δ became 1% or less. On average, over different values of RT parameters and $N_{0}$, each iMC simulation took about 0.2 s and solving $P_{s}(\lambda )$ per wavelength took about 160 s by executing the code on one GPU board (Nvidia, GeForce RTX 2080 Ti). The results of $P_{s}(\lambda )$ in Fig. 3 compare similarly to those obtained by methods using integrating sphere.^8^^,^^24^ For example, both of the range and wavelength dependence of $\mu_{s}$ and *g* agree well with those in Ref. 8 for the 20% intralipid sample while the range of $\mu_{a}$ is between those of Ref. 24 for 10% intralipid and Ref. 8. Since the 20% intralipid is high turbid with scattering albedo $a=\mu_{s}/\mu_{t}>98\%$ over the measured wavelengths, the large differences in the values of $\mu_{a}$ can be attributed to the very large errors in determining $\mu_{a}$ with values smaller than $\mu_{s}$ by three orders of magnitude as previously discussed.^8^ We note further that the values of optical thickness τ and albedo a of the intralipid sample reach the maximum values of 7.1 and 99.98%, respectively for λ=520  nm. Results of our study on samples of 20% intralipid with sufficiently large D values showed that current MPS method with the inverse solver reported here can yield unstable solutions of $P_{s}$ when the value of τ becomes larger than 7.1 for very large values of *a* above 99.9990%. A detailed comparison of the measured and calculated signals and analysis of background noise in signal measurement suggests that the instability of inverse solution is mainly caused by the variance in calculated signals, which may be mitigated without using $N_{0}$ much larger than those used for ISPs of τ≤7.1 for iMC simulations. A study is underway to further improve the PSO based inverse solver by taking into account the δ(P) distribution obtained from completed iterations, which is expected to enable accurate measurement of RT parameters for highly turbid samples with τ up to 15 without significant increase of computational cost. Such an improvement can make the approach of MPS concerned here capable of characterizing optically thick samples by their RT parameters for which the conventional approach with integrating sphere fails for inability to accurately measure collimated transmittance $T_{c}$.

## 4 Conclusions

We have analyzed the effect of variance by MC simulations on solving ISPs and proposed a novel objective function to reduce variance in calculated signals and speed up simulations. Combination of the objective function with the PSO algorithm leads to a rapid inverse solver for determination of RT parameters of turbid samples from three measured signals without integrating sphere. The inverse solver has been validated by the results of RT parameter retrieval on samples of 20% intralipid in a wavelength range of 520 to 1000 nm with ISPs solved rapidly using one GPU board. Taken together, we have demonstrated a new approach of MPS, which allows its translation into a powerful and easy-to-use instrument for clear separation and characterization of molecular composition and turbidity.

## Data Availability

All data in support of the findings of this paper are available within the article or as supplementary material. Code and other materials associated with this article are available upon request sent to the corresponding author.
