# Supplementary material for: Rapid inverse radiative transfer solver for multiparameter spectrophotometry without integrating sphere
Source: J Biomed Opt. 2024 Jan 2;29(Suppl 1):S11508. doi: 10.1117/1.JBO.29.S1.S11508 (PMC10759926; doi:10.1117/1.JBO.29.S1.S11508)
Supplement: Supplementary file 1 [file JBO_029_S11508_SD001.pdf]

Supplemental Materials for

Rapid inverse radiative transfer solver for multiparameter spectrophotometry without integrating sphere

Jiahong Jin[1,2,3], Zachary D. Jones[2], Jun Q. Lu[2,1] and Xin-Hua Hu [2,1,*]

[1]Institute for Advanced Optics, Hunan Institute of Science and Technology, Yueyang, Hunan 414006, China

[2]Department of Physics, East Carolina University, Greenville, NC 27858, U.S.A.

[3]School of Physics & Electronic Science, Hunan Institute of Science and Technology, Yueyang, Hunan 414006, China

* Corresponding authors: hux@ecu.edu

We have constructed a multiparameter system with detection configuration shown in Fig. 1 to acquire measured signals of $R_d(\lambda)$, $T_d(\lambda)$ and $T_f(\lambda)$ from a turbid sample. Figure S1 presents the signals measured from one 20% intralipid sample (I141-100ML, Sigma-Aldrich) of D = 102 μm in thickness. Other parameters of the optical layout as defined in Fig. 1 are given by: θ_0 = 8.5° for the incident beam angle; d_R = 34.4 mm and θ_R = 38° for the D_2 or R_d detector; d_T = 39.3 mm and θ_T = 39° for the D_3 or T_d detector; d_f = 63.6 mm and θ_f = 8.5° for the D_4 or T_f detector. The signal measurement was repeated by scanning wavelength λ from 520 to 1000 nm three times to obtain the mean and standard deviation values of these signals, which are shown by the symbols and error bars in Fig. S1. The measured signals were imported into the iMC code together to compare with calculated signals of R_{dc}, T_{dc} and T_{fc} and determined the RT parameters of the sample by the rapid inverse solver at each wavelength.

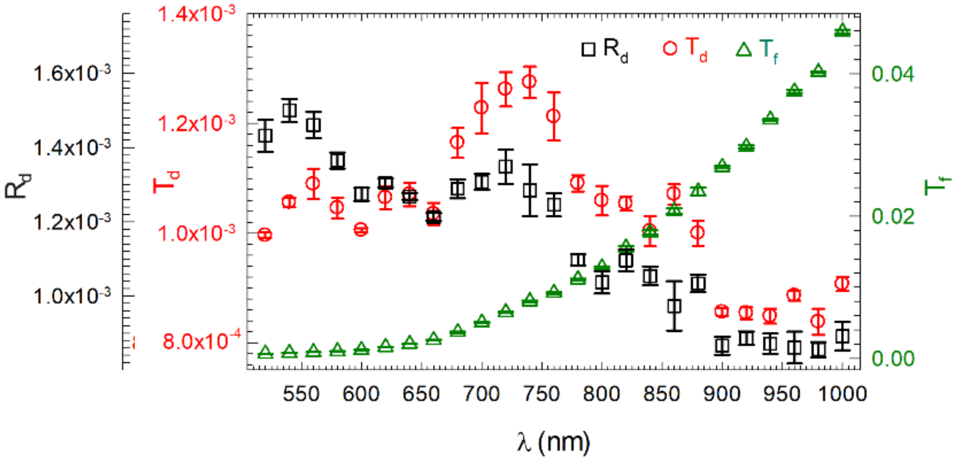

Fig. S1. The wavelength dependence of the measured signals of a 20% intralipid sample with thickness D = 102 μm. The symbols represent the mean values and error bars representing the standard deviation values of the signals obtained from 3 measurements.

We applied the interpolated values of real refractive index $n_r(\lambda)$ for 20% intralipid solution in signal calculation by iMC simulations, which were obtained by performing a cubic polynomial

fitting on the mean value of $n_r(\lambda)$ measured with s- and p-polarized incident beam {Ding, 2005 #110}. The measured data of $n_r(\lambda)$ and the fitting results are shown in Fig. S2.

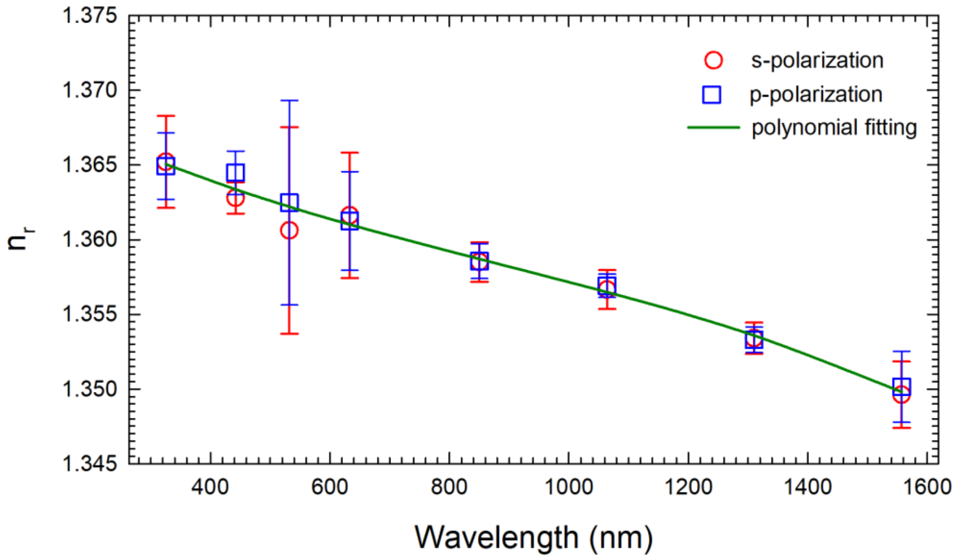

Fig. S2. The wavelength dependence of real refractive index n_r of 20% intralipid solution with experimental data represented by symbols and errors bars from {Ding, 2005 #110}. The solid line represents a cubic polynomial fitting of the mean values of n_r values with the s- and p-polarized incident beams.
